# Dynamic Progress in Technological Advances to Study Lipids in Aging: Challenges and Future Directions

**DOI:** 10.3389/fragi.2022.851073

**Published:** 2022-03-10

**Authors:** Fangyuan Gao, Emily Tom, Dorota Skowronska-Krawczyk

**Affiliations:** ^1^ Department of Ophthalmology, Center for Translational Vision Research, School of Medicine, UC Irvine, Irvine, CA, United States; ^2^ Department of Physiology and Biophysics, Department of Ophthalmology, Center for Translational Vision Research, School of Medicine, UC Irvine, Irvine, CA, United States

**Keywords:** lipidomic, technology, aging, lipids, review

## Abstract

Lipids participate in all cellular processes. Diverse methods have been developed to investigate lipid composition and distribution in biological samples to understand the effect of lipids across an organism’s lifespan. Here, we summarize the advanced techniques for studying lipids, including mass spectrometry-based lipidomics, lipid imaging, chemical-based lipid analysis and lipid engineering and their advantages. We further discuss the limitation of the current methods to gain an in-depth knowledge of the role of lipids in aging, and the possibility of lipid-based therapy in aging-related diseases.

## Introduction

Aging is a complex, multifactorial process that is characterized by a gradual decline in many physiological functions at the cellular and organismal levels. These nonmonotonic changes occur at varying rates across different species, spatially—among tissues within an individual, and temporally—at different timepoints in an organism’s lifespan. Epigenetic, transcriptomic and proteomic studies have shown clear evidence for spatial heterogeneity of aging rates among tissues (Liu et al., 2010; Simon et al., 2013; Consortium, 2020; Moaddel et al., 2021). However, there is a considerable gap in comparable efforts to resolve the aging lipidome, or lipid profile, at cellular, tissue, or organismal levels. Changes in the lipidome during lifespan have been measured across several tissues, which points to opportunity for exploration of this understudied class of biomolecules (Almeida et al., 2021). Many recent efforts have focused on the aging lipidome of the central nervous system (CNS), the second-most lipid-rich structure (after adipose tissue), due to its unique lipid composition (Jové et al., 2021). For example, approximately 700 lipid species were quantified and evaluated from mouse brains during aging and pathological conditions to identify cell-type and region-specific lipid profiles (Fitzner et al., 2020). Similarly, the lipid composition of the eye, another part of the CNS, undergoes changes related to age and disease state (Liu et al., 2010; Gorusupudi et al., 2016; Chen et al., 2020a; Lewandowski et al., 2021a).

Lipids play a key role in many biological processes, such as cellular structure, energy storage, and cell signaling, and are essential for the maintenance of cellular homeostasis. Their dysregulation is associated with a variety of health conditions, including diseases and aging. Altered lipid metabolism results in changes in the membrane lipid environment, such as an age-related increased polyunsaturated fatty acid (PUFA) to monounsaturated fatty acid (MUFA) ratio, increased ceramide levels and decreased cholesterol levels (Skowronska-Krawczyk and Budin, 2020; Lewandowski et al., 2021b; Mutlu et al., 2021). While harmful lipid accumulation and peroxidation have been correlated with aging, emerging studies have only begun to explore the mechanistic link between lipid metabolism and pro-longevity signaling pathways (Han et al., 2017; Qi et al., 2017).

Multiple epidemiological and genetic studies show unequivocally that lipidomics has great potential in revealing new biology not captured by traditional lipids and lipoprotein measurements. Lipid species measurements, like other intermediate phenotypes, increases statistical power to detect genetic associations and hence provide opportunity to discover new lipid loci. When combined with genome-wide association studies (GWAS), human lipidome profiles have unparalleled potential to discover genetic variants linked to traditional blood lipids—low-density lipoprotein cholesterol (LDL-C), high-density lipoprotein cholesterol (HDL-C), and triglycerides—and established functional links between lipid levels and disease (Ference et al., 2017). Genetic discovery on more than 1.65 million individuals from five ancestry groups improved fine-mapping functional variants that contribute to lipid-level variations and polygenic prediction scores for increased LDL-C levels and cardiovascular conditions (Graham et al., 2021). Recently, these GWAS studies have been expanded to include a greater set of lipid species (Tabassum et al., 2019; Tabassum and Ripatti, 2021), covering the major glycerophospholipid, sphingolipid, glycerolipid, sterol and fatty acyl classes in serum and plasma samples (Cadby et al., 2020). Integration of lipidome, genome and phenome already revealed genetic regulation of lipidome and identified novel risk loci of cardiovascular disease (CVD) beyond standard lipid profiling of traditional lipids (Tabassum et al., 2019). The findings from these studies hold great potential for the future of preventive and precision medicine.

**FIGURE 1:**
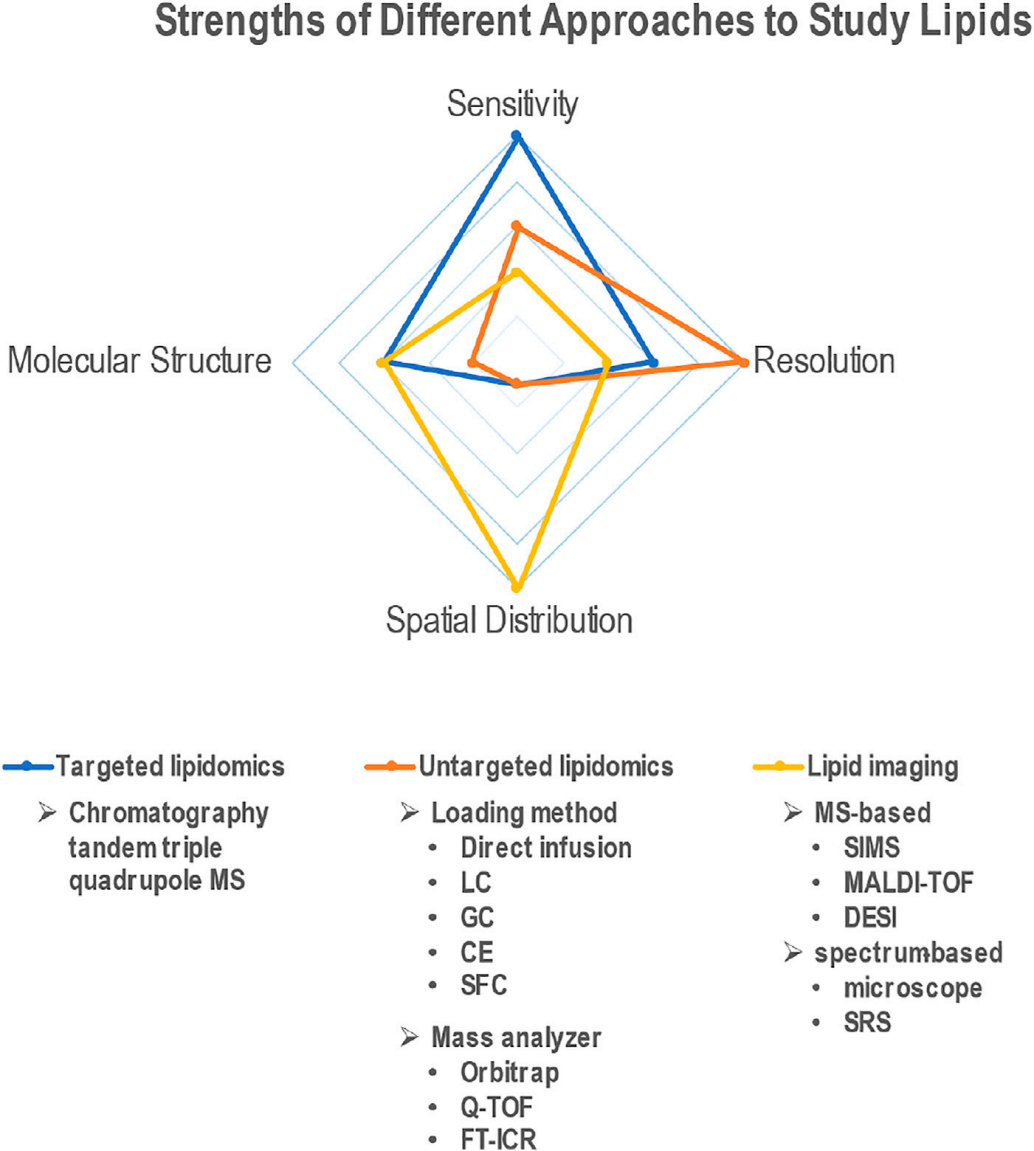
Radar plot depicting the relative advantages of different techniques (targeted lipidomics, untargeted lipidomics and lipid imaging) to study lipids. Comparison criteria include ability to quantify lipids (sensitivity), identify different lipid species (resolution), discern molecular structure, and measure spatial distribution across tissues.

Recent advances in technologies that can be used to analyze lipid composition, structure and localization have been instrumental in understanding the role of these important molecules in aging processes (Figure 1). For example, in a recent study, ultra-performance liquid chromatography to quadrupole time-of-flight mass spectrometry (UPLC-Q-TOF-MS) based lipidomic analyses revealed the role of fatty acid binding protein 3 (FABP3) in altering membrane lipid composition and inducing ER stress in aged muscle (Lee et al., 2020). Also, altered membrane lipid composition, and more specifically, decreased membrane fluidity, have been implicated in aging. For instance, in rat primary cortical neurons treated with hydroxyurea to provoke senescent-like alterations, changes in membrane lipid composition led to decreased membrane fluidity (Yu and Cheng, 2021). In another study, using both ensemble and single-molecule fluorescence imaging techniques, decreased membrane fluidity and increased membrane hydrophobicity were measured in senescent cells (Wi et al., 2021). Interestingly, through metabolic engineering of unsaturated lipid biosynthesis, the physiological effects of increased membrane viscosity on fundamental eukaryotic processes such as respiratory metabolism were investigated (Budin et al., 2018). In a study from our laboratory, CRISPR-Cas9 based genome editing technology was used to generate mutant mice containing a point mutation in *Elovl2* (Elongation of Very Long Chain Fatty Acids-Like 2), whose promoter region is increasingly methylated with age. This mutation caused changes in the lipid composition across several tissues, including the liver and retina, and resulted in an accelerated aging phenotype and premature visual impairment (Chen et al., 2020b). Despite these advances in knowledge, the field of lipid research faces several challenges including detection sensitivity, resolution, and identification.

Another challenge to studying lipids is the shortage of imaging techniques that do not require cells to be fixed and processed. Fluorescent dyes, such as Nile Red, BODIPY, Oil Red O and Sudan III, have been used to visualize intracellular neutral lipids, but possess several drawbacks including limited photostability and small Stokes shift, causing cross-talk between the excitation source and the fluorescence emission (Fam et al., 2018). Recently, solvatochromic coumarin derivatives were synthesized for selective live-cell imaging of intracellular lipid droplets (Jana et al., 2020), which have been shown to accumulate in the aging brain (Marschallinger et al., 2020).

Although they are a major class of biological molecules, lipids have not been studied as well as proteins, partly due to technological limitations. Recent eruption of novel or improved tools has allowed the field to formulate new hypotheses and provide more detailed answers. Targeted/untargeted lipidomics, mass spectrometry/spectra-based lipid imaging, lipids-functionalized reporters are accelerating the rate of discoveries in lipid biology. Here, we will review available techniques to studying lipids in aging and highlight several of the recent technological advances in lipid biology that have allowed us to better visualize and analyze lipids *in vivo*. Finally, we will discuss the current challenges in analyzing role of lipids in aging.

## Lipidomics

Lipidomics is a discipline that studies cellular lipids on a large scale based on analytical chemistry principles and technological tools, particularly mass spectrometry (MS). Due to the compatibility with multiple common separation methods, including liquid chromatogram (LC), gas chromatogram (GC), capillary electrochromatography (CE) and emerging supercritical fluid chromatography (SFC), availability of different ionization methods and mass analyzer types, MS-based approaches offer high sensitivity and high resolution on a broad application range (Figure 1). The two main lipidomic approaches include targeted and untargeted lipidomics. Targeted lipidomics mainly depends on high-sensitivity MS, such as triple quadrupole mass spectrometer (Takeda et al., 2018), while untargeted lipidomics relies on high-resolution MS, such as Q-TOF(Yan et al., 2021), orbitrap mass spectrometer (Taguchi and Ishikawa, 2010) and Fourier transform ion cyclotron resonance (FT-ICR) (Haler et al., 2019). Electrospray ionization (ESI) is the most used ionization approach for lipidomics. Depending on charge states and fragmentation, different lipid classes need to be scanned in different modes. For phospholipids, data are collected in ESI positive (+) and negative (−) ionization modes in separate runs. Free fatty acids are usually detected in negative mode, and positive mode is usually better for cholesterol, CERs, sphingomyelin (SM) (Cífková et al., 2015), glycerolipids (GL), glycerophospholipids (GP), and sphingolipids (SP) (Shaner et al., 2009; Della Corte et al., 2015). Most neutral lipids, such as CEs, TAGs, wax esters (WEs) (Iven et al., 2013), can be detected in positive mode. An alternative way to ionize neutral lipids is atmospheric pressure chemical ionization (APCI), which is suitable for nonpolar and low-polar compounds. APCI-MS has been used for identification and quantification of TAGs (Lísa et al., 2009), WEs (Vrkoslav et al., 2011), and fatty acid methyl esters (Vrkoslav et al., 2020). In addition, matrix-assisted laser desorption/ionization time of flight mass spectrometry (MALDI-TOF MS) can also be used to characterize WEs (Vrkoslav et al., 2009) and TAGs (Pannkuk et al., 2012).

The purpose of untargeted lipidomics is to identify and quantify as many molecular species of lipids as possible from biological samples. Precursor ions and fragment ions produced by high-energy collision-induced dissociation (HCD) are utilized to examine the lipid compositions. For example, cholesterol generates an intense fragment ion peak at m/z 369 ([M + H–H_2_O]^+^) (Liebisch et al., 2006). Precursor ion scans of m/z 184 commonly employed for the identification of choline containing species, including phosphatidylcholine (PC) and SM (Taguchi et al., 2005). Different neutral losses have been identified for different phospholipids. Neutral loss occurs in MS when all charge is retained on one of the precursor fragments, resulting in a neutral product (Martin et al., 2005). For example, neutral loss of 141, 185, 189 and 277 Da (Dalton) were observed for phosphatidylethanolamine (PE), phosphatidylserine (PS), phosphatidylinositol (PI) and phosphatidylglycerol (PG), respectively (Taguchi et al., 2005; Schwudke et al., 2006). Product ions arising by neutral loss of 44 Da were observed with FA (Kerwin et al., 1996). Neutral losses of 78, 98, and 136 Da were observed for long-chain polyunsaturated fatty acids with five or more double bonds (Thomas et al., 2012).

In untargeted lipidomics, the samples are delivered to MS through direct infusion mode (shotgun lipidomics) or after chromatography separation. Both methods have their own advantages and disadvantages. Shotgun lipidomics is a rapid way for lipid analysis, and it ensures that the matrix is consistent for all lipids (Wang and Han, 2019). LC-MS-based untargeted lipidomics provides a way to avoid ion suppression, and therefore, potentially has higher sensitivity (Lísa and Holčapek, 2015). Data-dependent acquisition (DDA) is a more common fragmentation method in LC-MS-based untargeted lipidomics (compared to Data-independent acquisition, DIA). Only precursor ions that have relatively high abundance (above ion intensity threshold) are applied by different collision energies (Monnin et al., 2018). Contrary to the “absolute” quantification in targeted lipidomics, which relied on internal or external standards for quantification, untargeted lipidomics provides quantitation by statistical analysis using either raw or normalized peak intensities and compared across groups of samples (Cajka et al., 2017).

Depending on instrumentation, acquisition mode, mass analysis routine, and data format, multiple software and platforms were developed for lipidomics data analysis, including LIPID MAPS (Fahy et al., 2007), LipidSearch (Taguchi and Ishikawa, 2010), LipidView (Ejsing et al., 2006), Lipidblast (Kind et al., 2013) and LipidXplorer (Herzog et al., 2012), LipidFinder (O’Connor et al., 2017) and others. The choice of the platform can be driven by the particular type of analysis as well as by the simple accessibility. The number of available ways to analyze data is overwhelming, and different methods have their own pros and cons. Therefore it is of highest importance to use the most suitable methods for data acquisition and normalization. We are currently in the midst of standardizing the technical and analytical approaches. In addition, concerted efforts of many laboratories in finding common grounds related to the lipid terminology (Liebisch et al., 2020) should help researchers to understand their data in the context of data of other scientists.

The development of novel techniques and instruments have made it possible to maximize the coverage of lipid species detected and quantified in complex biological samples, such as plasma and different tissues (Contrepois et al., 2018; Naoe et al., 2019). For example, combined with droplet extraction technique and a pulsed direct current electrospray ionization, MS has been applied to detect metabolites from a single cell on QE-Orbitrap mass spectrometer, identifying more than 300 phospholipids (Zhang et al., 2018).

## Lipid Imaging

With the development of the high–resolution, high-sensitization mass spectrometry, mass spectrometry-based imaging (MSI) has been successfully used to map the distribution of various lipid species from biological surfaces of different tissues and organs and showed potential for diagnosis and prognosis of diseases (Zhao et al., 2020; Patterson et al., 2016). Current MSI techniques include secondary ion mass spectrometry (SIMS), matrix-assisted laser desorption ionization (MALDI) and desorption electrospray ionization (DESI) (Figure 1). Among them, MALDI-MSI is the most developed technique and therefore, is currently the most commonly used. It shows the potential to provide a vast amount of information on the abundances of specific lipids, but also provides direct detection and spatial distributions of molecules within biological tissues. Several laboratories have used MALDI-MSI to understand the lipid composition of the retina, most notably in the photoreceptor OS of human retinas (Zemski Berry et al., 2014; Anderson et al., 2020). Changes in lipid composition upon disease or mutations have also been recently studied (Kautzmann et al., 2020). However, MS-based imaging methods cannot determine either the exact location of lipid in certain cellular organelles *in situ*, or image lipids on single cells due to the low spatial resolution (10–100 μm) and insufficient imaging depth (section thickness). However, powerful imaging and statistical software have increased the performance of MSI. For example, SpaceM, a method for detection of metabolites at the single-cell level *in situ* by using MALDI-MSI integrated with light microscopy and digital image processing, allows achievement of subcellular precision, high throughput metabolomics imaging (Rappez et al., 2021). A dedicated program was written to allow the conversion of the mass spectra into a format compatible with the Biomap software and to help DESI imaging of direct analysis of brain tumors (Eberlin et al., 2012).

Different from MSI, spectral imaging, such as Stimulated Raman Scattering (SRS), has a deeper imaging depth than other methods (up to 1 mm) allowing for 3D volumetric imaging (Figure 1) (Wei et al., 2019). SRS works by detecting the vibration of chemical bonds in biological tissues, exploiting the fact that different chemical bonds vibrate at distinct frequencies (Downes, 2015). Each molecule understudy has its own vibrational spectrum profile, allowing SRS imaging to be chemically specific. Compared with spontaneous Raman spectroscopy, SRS microscopy offers at least 1,000-fold faster acquisition (Fung and Shi, 2020; Shi et al., 2021). Moreover, SRS can be used for 3D optical sectioning even in living animals (Shi et al., 2018). Finally, SRS signal intensity is linearly proportional to the concentration of a chemical bond, which can thus allow quantitative imaging (Li et al., 2018a). Shi et al. (Shi et al., 2018) developed a platform that combined deuterium oxide (D2O) probing with SRS (DO-SRS) microscopy to image *in situ* metabolic dynamics of proteins, lipids, and DNA in a variety of model organisms, including fibroblast-like COS7 cells, *C. elegans* larva, zebrafish embryos, and mouse tissues, exhibiting powerful ability to visualize lipogenesis and protein synthesis.

Rapid development of solvatochromic dyes in the last decade have allowed us to better visualize plasma membranes (Danylchuk et al., 2020) and intracellular organelle membranes (Niko et al., 2016) in living cells. Solvatochromic dyes are fluorescent probes that can alter their fluorescence wavelength in response to the polarity of their environment, which is especially useful in studying the heterogeneity of lipid biomembranes. Unlike “classical” dyes, such as rhodamine and cyanines, the environment-sensitive solvatochromic dyes are generally push-pull fluorophores that undergo intramolecular charge transfer (ICT) upon photoexcitation (Kucherak et al., 2010). These probes can also be derivatized to target specific organelles, which can then be used to compare lipid profiles and changes in lipid order of intracellular compartments in response to external stress (Danylchuk et al., 2021).

## Chemical-Based Lipid Analysis

Chemical approaches provide a sensitive and flexible way to understand the roles of lipids in biology. Radioisotopes labeled lipid precursors (e.g., 14C, 32P for polar lipids) have been previously used to map the lipids synthesis pathway (Moldoveanu and Kates, 1988). Now, fluorescently-labeled lipids combined with super-resolution microscopy enable high-resolution visualization of lipids. Photoactivatable lipid probes are powerful tools for lipid biology studies through light-controlled imaging at high spatial and temporal resolution (Mentel et al., 2011; Haberkant et al., 2013). So far, it has been used for imaging of lipid droplets in live cells (Gao et al., 2017; Kavunja et al., 2020), membrane interface-protein interactions, and organelle-specific imaging (Li et al., 2018b).

To improve the sensitivity of MS-based lipids detection, different derivatizations are needed for different lipid classes. For example, the most common quantification of fatty acids was performed by GC-MS after methyl ester derivatization. Alternate ways have been developed to improve the quantification of fatty acids by LC-MS, such as derivatization by precharged quaternary ammonium salt of the trimethylaminoethyl ester (TMAE) (Li and Franke, 2011). Derivatizations also help to reveal the lipid structure when targeted to different groups, such as carbon-carbon double bond and carboxyl group (Wang et al., 2013; Unsihuay et al., 2021). For example, photochemical derivatization has been used to identify C=C location and relatively quantify *sn*-position isomers within single-cell (Li et al., 2021).

## Lipid Engineering

Over the past several decades, complementary synthetic biology and metabolic engineering techniques have been applied to functional studies of lipids (Moore et al., 2021). Nano-injection of lipids has been successful in directly delivering different phospholipids, including PE, PC, and LPC, into intracellular membranes (Aref et al., 2020). Powerful genetic tools, such as CRISPR-Cas9 mediated knockouts, have created programmable, robust and versatile methods for genome editing across several organisms (Chen et al., 2020b; Dasyani et al., 2020). However, classical approaches offer only binary or on/off control of gene expression. In contrast to proteins and nucleic acids, lipids are not direct gene products; rather, they are synthesized via many complex metabolic pathways. Therefore, precise manipulation of genes involved in lipid metabolic pathways is necessary to control lipid stoichiometry in living cells. Titratable expression platforms, such as the Tet repressor or pBAD promotor, allow for such fine-tuning of gene expression. Using this approach, cellular respiration in *E. coli* was measured as a function of unsaturated fatty acid (UFA) levels in the inner membrane (Budin et al., 2018). Bidirectional titration of gene expression in a metabolism-wide manner has also been achieved by integrating plasmid-based single-guide RNA (sgRNA) library methodology with a CRISPR-dCas9 system (Bowman et al., 2020).

## Future Directions

Although mass spectrometry is extremely powerful for analysis of different lipid species, there are still challenges to identify all the lipids. Some lipids with multi-phosphate groups, such as phosphoinositide, need to be derivatized to improve the sensitivity of detection due to their low ionization efficiency. Lipids containing super-long-fatty-acid are difficult to ionize or vaporize; therefore, it is challenging to analyze them. The discrimination of isomers of lipids and the localization of the double bonds in lipids are other challenges for most mass spectrometry.

There is no doubt that the lipid composition of membranes directly and indirectly influences the activity of receptors, channels, and other transmembrane and membrane-associated proteins. However, the molecular role of membrane lipids is still not fully determined. Due to the limited detection sensitivity and insufficient depth of MS-based imaging techniques, detailed mapping of region-specific lipid profiles in tissues, such as the heterogeneous distribution of cholesterol in rod outer segments, is also daring. To understand the interaction between lipids and associated proteins, methods for isolating lipid-protein complexes, detecting lipid-protein interactions and combining muti-omics data need to be developed. A series of interdisciplinary studies involving biophysics of membranes, biochemistry, and molecular biology will be required to understand the regulation of protein activity by membrane lipids and how to modulate them. One challenge is the ability to purify membrane proteins in their native lipid environment to analyze protein-lipid functional and biophysical interactions. The conventional method of using detergents to isolate membrane proteins does not accurately preserve the surrounding membrane bilayer, which may influence both protein structure and function. The emergence of styrene maleic-acid lipid particles (SMALPs) has been developed to extract the membrane bilayer containing proteins into discrete nanodiscs, which can then be subjected to purification methods such as immunoaffinity chromatography. Using this technology, major differences in lipid composition between the central and rim regions of rod outer segment discs were described (Sander et al., 2021). The same methods can be used *in vitro* to study the impact of changes in lipid bilayer composition on receptor activity (Grime et al., 2021; Szundi et al., 2021).

With our current knowledge in hand, it has been possible to use analog control of lipid biosynthesis using highly titratable expression platforms (Budin et al., 2018). However, further development of techniques is required to significantly improve the understanding of the role of lipids in biology and aging. Future directions include novel methods of ionization, super-resolution imaging, better understanding of lipid metabolism, and modifying the lipid composition of specific organelle membranes.

While advances in lipidomics, one of the younger members of the “omics family”, have made it possible to measure changes in the aging lipidome, mechanistic understanding of these changes remains a challenge. Recent advances in genome mapping and molecular biology have begun to connect the genes involved in lipid biosynthesis and their functions. Together with emerging genomics tools, lipidomics has the potential to identify lipid-modulating genetic variants in aging and various diseases, providing us with many new opportunities to develop better predictive and personalized medicine. Looking forward, interdisciplinary approaches and multilaboratory collaborations will shed light on the functions of this dynamic class of biomolecules and reveal the potential for lipid-based therapies in age-related diseases.
